# Development of *Tg(UAS:SEC-Hsa.ANXA5-YFP,myl7:RFP)*; *Casper(*roy^−/−^,nacre^−/−^*)* Transparent Transgenic In Vivo Zebrafish Model to Study the Cardiomyocyte Function

**DOI:** 10.3390/cells10081963

**Published:** 2021-08-02

**Authors:** Surendra K. Rajpurohit, Aaron Gopal, May Ye Mon, Nikhil G. Patel, Vishal Arora

**Affiliations:** 1 Georgia Cancer Center, Medical College of Georgia, Augusta University, Augusta, GA 30912, USA; MYEMON@augusta.edu; 2 Department of Medicine, Division of Cardiology, Medical College of Georgia, Augusta University, Augusta, GA 30912, USA; AGOPAL@augusta.edu; 3 Department of Pathology, Medical College of Georgia, Augusta University, Augusta, GA 30912, USA; NPATEL4@augusta.edu

**Keywords:** zebrafish, cellular phenotype, transparent skin mutant-*Casper(*roy^−/−^,nacre^−/−^*)*, transgenic strain, cardiomyocyte, annexin-5, cellular phenotype, in vivo confocal imaging, fluorescent screening

## Abstract

The zebrafish provided an excellent platform to study the genetic and molecular approach of cellular phenotype-based cardiac research. We designed a novel protocol to develop the transparent transgenic zebrafish model to study annexin-5 activity in the cardiovascular function by generating homozygous transparent skin *Casper(*roy^−/−^,nacre^−/−^*)*; myl7:RFP; annexin-5:YFP transgenic zebrafish. The skin pigmentation background of any vertebrate model organism is a major obstruction for in vivo confocal imaging to study the transgenic cellular phenotype-based study. By developing *Casper(*roy^−/−^,nacre^−/−^*)*; myl7; annexin-5 transparent transgenic zebrafish strain, we established time-lapse in vivo confocal microscopy to study cellular phenotype/pathologies of cardiomyocytes over time to quantify changes in cardiomyocyte morphology and function over time, comparing control and cardiac injury and cardio-oncology. Casper contributes to the study by integrating a transparent characteristic in adult zebrafish that allows for simpler transparent visualization and observation. The *Casper(*roy^−/−^,nacre^−/−^*)* transgenic progenies developed through cross-breeding with the transgenic strain of *Tg(UAS:SEC-Hsa.ANXA5-YFP,myl7:RFP)*. Confocal and fluorescent microscopy were being used to obtain accurate, precise imaging and to determine fluorescent protein being activated. This study protocol was conducted under two sections; 1.1: Generation of homozygous *Tg(UAS:SEC-Hsa.ANXA5-YFP,myl7:RFP)*; *Casper(*roy^−/−^,nacre^−/−^*)* zebrafish (generation F01-F06) and 1.2: Screening and sorting the transparent transgenic progeny and in vivo imaging to validate cardiac morphology through in vivo confocal imaging. We coined the newly developed strain as *Tg(UAS:SEC-Hsa.ANXA5-YFP,myl7:RFP)*; *Casper(*roy^−/−^,nacre^−/−^*)^gmc1^*. Thus, the newly developed strain maintains transparency of the skin throughout the entire life of zebrafish and is capable of application of a non-invasive in vivo imaging process. These novel results provide an in vivo whole organism-based platform to design high-throughput screening and establish a new horizon for drug discovery in cardiac cell death and cardio-oncology therapeutics and treatment.

## 1. Introduction

The heart is the first organ to form and function in the embryo, and its formation is followed by a series of vital events in the life of the organism. Cardiac malformation at this stage is the most common form of human birth defect, and abnormalities of the adult heart threaten to be the most prevalent cause of morbidity and mortality in the modern era. In the United States, heart failure is as epidemic and nearly a million new cases are diagnosed annually [1]. The early 21st century has marked a transition from a physiological and functional approach towards the heart to a deeper understanding of cardiac function at the genetic and molecular levels. These discoveries have provided new therapeutic dimensions for palliation and prevention of heart pathophysiology, as well as having raised new questions, challenges, and pathways for the next generation of cardiac research. Advances in cardiology will be driven by the integration of new technologies and modeling systems. The zebrafish (*Danio rerio*) model has provided an excellent platform to study the genetic and molecular approach of basic and translational cardiac research. The genetically engineered zebrafish model has been established and applied in human congenital and acquired cardiac disease research [2]. Furthermore, the zebrafish model has potential to be used as a genetic screen for mutations that affect cardiac regeneration and is an opportunity to explore the functions of genes that control cardiac dynamics. It may also accelerate new therapeutic strategies [3]. Zebrafish heart cells are similar to human heart cells at the molecular level and determine the function of genes that control cardiac function and dysfunction. In zebrafish, the cardiac myocyte-specific gene *myl7* is a myosin, light chain 7, regulatory gene that has calcium binding activity. In the zebrafish embryonic somitogenesis developmental stage, known as cardiogenic differentiation, expression of sarcomeric myosin genes is observed in the anterior lateral plate mesoderm (ALPM) as early as the 14-somite stage [4,5,6]. Expression of myosin light chain polypeptide 7, *myl7*, formerly indicated as cmlc2, is initiated in few cells, with the number of cells expressing myl7 increasing over time. This gene is involved in several processes, including cardiac muscle cell proliferation, heart contraction, and myofibril assembly. It is orthologous to human *MYL7* gene (myosin light chain 7) and mouse *Myl7*. In zebrafish, myl7 is a myosin light chain 7 gene and has been identified as a regulatory gene of heart orthologs to human MYL7 myosin light chain 7 [6,7,8].

In the heart, annexin-5 activities contribute to cardiomyocyte dedifferentiation, proliferation, and epicardial injury responses which lead to cardiac cell death by apoptosis and narcosis pathways. To determine the cell death pattern of cardiomyocytes by studying apoptosis, necrosis, and scar formation, we planned to use the annexin-5 transgenic line generated by Dr Peterson’s lab. To visualize the dying cell in vivo, a transgenic line expressing an annexin A-5-YFP fusion was created [9]. The A5-YFP fusion function is to detect cells expressing phosphatidylserine (PS) on the cell surface, a marker for apoptotic and other forms of cell death. Cardiac Myocyte AnnexinA5 Strain: Dr. Peterson’s lab has generated *Tg(UAS:SEC-Hsa.ANXA5-YFP,myl7:RFP)f12* strain where *SEC* is a short signal peptide that was used to cause secretion of the ANX5A protein. The founder line of *Tg(UAS:SEC-Hsa.ANXA5-YFP,myl7:RFP)f12* gifted to us by Dr Peterson’s lab.

In vertebrates, including zebrafish, murine, and human systems, the in vivo spatial resolution is limited due to the normal opacification of skin and subdermal structures. For in vivo imaging, the skin transparency is the primary requirement and, in order to maintain the transparency, pigmentation must be blocked. Blocking of the pigmentation can be maintained by chemical inhibition by blocking melanization. Chemical inhibitor PTU (1-phenyl 2-thiourea) is adequate to block the pigmentation in pigment epithelium melanization [10]. Chemical inhibition treatment is temporary and possible until the organism is treated with the chemical inhibitor agent. The zebrafish *Casper(*roy^−/−^,nacre^−/−^*)* mutant maintains transparency throughout its life and serves as an ideal combination of sensitivity and resolution for in vivo stem cell analyses and in vivo imaging [11]. Since the *Casper(*roy^−/−^,nacre^−/−^*)* was named as Casper by the founder, we used the Casper name for this mutant line in the text of the manuscript. The *Casper(*roy^−/−^,nacre^−/−^*)* transparent mutant line was gifted by Dr Zon’s Lab.

Our main aim is to develop the annexin-5 activity in the cardiovascular function under normal and in metabolically aberrant conditions by developing homozygous *Tg(UAS:SEC-Hsa.ANXA5-YFP,myl7:RFP)*; *Casper(*roy^−/−^,nacre^−/−^*)* transparent transgenic zebrafish. By developing a *Casper/myl7/Annexin-5* transparent transgenic zebrafish model, we established time-lapse in vivo confocal microscopy to study cellular phenotype and pathologies of the cardiomyocytes over time in a newly developed transparent transgenic zebrafish strain to quantify changes in cardiomyocyte morphology and function overtime, comparing control and cardiac injury models [10,12,13]. We developed Casper transgenic progenies through cross breeding with the transgenic strain of *Tg(UAS:SEC-Hsa.ANXA5-YFP,myl7:RFP)*. Confocal and fluorescent microscopy were used to image and determine the fluorescent protein being activated. We conducted our study under two sections; 1.1: Generation of homozygous *Tg(UAS:SEC-Hsa.ANXA5-YFP,myl7:RFP)*; *Casper(*roy^−/−^,nacre^−/−^*)* (Generations F01-F06) and 1.2: Screening and sorting of transparent transgenic progeny and in vivo imaging to validate cardiac morphology through in vivo confocal imaging. We named the newly developed strain as *Tg(UAS:SEC-Hsa.ANXA5-YFP,myl7:RFP)*; *Casper(*roy^−/−^,nacre^−/−^*)^gmc1^*. The newly developed zebrafish strain could expedite development of treatment for cardiovascular diseases. Our approach could yield crucial new insights into in vivo cardiomyocyte imaging via confocal microscopy and could track the cell death pattern in cardiomyocytes, leading to the development of novel therapeutic approaches.

## 2. Materials and Methods

### 2.1. Fish Housing

Our laboratory maintained the stocks of the zebrafish strains at the Augusta University Transgenic Zebrafish Core Facility. All fish progenies housing was in tanks of appropriate dimensions regarding size and density, with optimal biological and water filtration by maintaining water pH, conductivity, and *UV-*light system to prevent bacterial and fungal growth. Embryos, larvae, and adult zebrafish were maintained at 28.5 °C with a consistent 14:10 h light-dark cycle. An appropriate feeding regimen was provided and maintained to promote robust mating. All zebrafish experiments were performed in accordance with the relevant guidelines and regulations and approved from the Augusta University Animal Care and Use Committee (IACUC).

### 2.2. Zebrafish Lines

**Figure 1:**
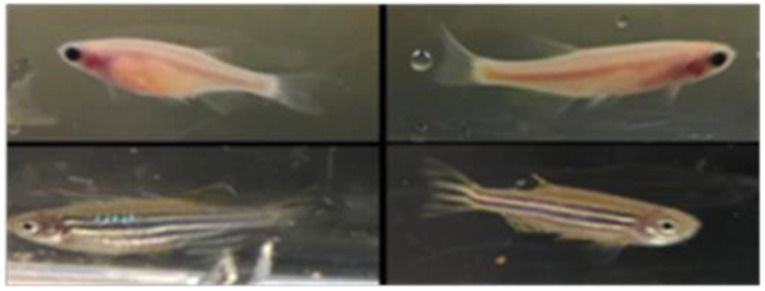
The zebrafish that portray the wild-type pigmentation (stripes). The right panels are the male zebrafish (darker blue stripes) and the left panels are the female zebrafish (rounder belly). Upper panels are *Casper(*roy^−/−^,nacre^−/−^*)* fish and lower panels are transgenic lines; cardiomyocyte/annexin-5 transgenic *Tg(UAS:SEC-Hsa.ANXA5-YFP,myl7:RFP)* having normal skin pigmentation pattern.

The following fish strains were used: wild type; WT-AB, transgenic lines; cardiomyocyte/annexin-5 transgenic *Tg(UAS:SEC-Hsa.ANXA5-YFP,myl7:RFP)f12* [9], and transparent skin mutant line “*Casper(*roy^−/−^,nacre^−/−^*)*” [11] (Figure 1).

### 2.3. Equipment

#### 2.3.1. Fluorescent Microscope

Keyece fluorescent microscope (model BZX-800) used for screening of the zebrafish larvae at 72 hpf stage to confirm the transgenic expression.

#### 2.3.2. Confocal Microscope

A Leica Confocal microscope (model: Leica Stellaris-5 Microsystem) was used for in vivo imaging of zebrafish larvae at 72 hpf (hours post-fertilization) stage. The in vivo image of the transgenic zebrafish larvae was captured and analyzed by LAS-X software.

### 2.4. Chemicals and Reagents

#### 2.4.1. Fish Water

The fish water obtained from the Zebrafish Core Facility Water Circulating System for fish units and was used to prepare the media for larvae. Prior to use, the fish water was properly tested and compared with the 1× E3 media. (A combination of 29.22 g of NaCl, 1.27 g of KCl, 3.33 g of CaCl_2_, and 3.97 g of MgSO4 was used to produce 1 L of 100× E3 medium). The medium was placed at room temperature (RT: 21 °C) for 1 h on a stir plate. The pH was adjusted to 7.4 with NaOH. Stocks of 100× E3 may be stored at RT for 3 months or at 4 °C for 6 months. In order to prepare working 1× E3 medium, 100× E3 medium diluted (above) to 1× in water. The final 1× E3 solute concentration was as follows: NaCl (5 mM), KCl (0.17 mM), CaCl_2_ (0.3 mM), and MgSO4 (0.33 mM). Stocks of 1× E3 may be stored at RT (room temperature) for 3 months.

#### 2.4.2. Phenyl-2-thiourea (PTU)

Phenyl-2-thiourea (PTU) (Acros Organics, (cat. no. 103-85-5)) is highly toxic; so appropriate PPE was worn when handling the compound. For the 50× PTU stock solution, 1.520 g of PTU was added to 1 L of 1× E3 medium. The solution was mixed on a hotplate at 50 °C for 2 h (protected from light). The solution was cooled to RT, and then aliquots were prepared in a fume hood. Aliquoted PTU stock solution can be stored at −20 °C for 1 year (protected from light). For the 1× PTU solution, 50× PTU stock solution (above) was diluted to 1× with 1× E3. This solution can be stored at RT for 3 months [10].

#### 2.4.3. Clove Oil

Clove oil (Sigma-Aldrich, cat. no. C-8392) is toxic when inhaled, and it may cause skin/eye irritation; therefore, appropriate PPE was worn when handling the compound. Clove oil was used to anesthesia the larvae for experimental purpose, using a 34× clove oil anesthetic solution. A 0.68% (*v*/*v*) clove oil solution was prepared in fish water. This was diluted to a 0.02% working solution in fish water. The working solutions were made freshly from the stock solution for each assay [10].

#### 2.4.4. Agarose

A 1.6% agarose solution (Sigma) was prepared in fish water.

### 2.5. Methodology

#### 2.5.1. Pigmentation Blockage by Chemical Inhibition

PTU (1-phenyl 2-thiourea) is a tyrosinase inhibitor used to block melanization. The transparency of the newly generated progeny was maintained through blocking the pigmentation through chemical inhibition. The zebrafish embryos started to develop the skin melanophore pigmentation from 14 hpf, which continued throughout the lifespan of the fish. For imaging purposes, to screen the fluorescent expression, the embryos and larvae were treated with the PTU at 12 hpf to block pigmentation deposition at the concentration of 0.003% embryonic media (EM).

#### 2.5.2. Stress/Pain Management

All stress and/or pain-inducing manipulations (screening via microscope and imaging by confocal microscopy) were carried out in the presence of clove oil working solution. Protocols for reducing stress during fluorescent and confocal microscopy procedures (dimmed light, reduced noise, etc.) were implemented to avoid any discomfort associated with the process.

#### 2.5.3. In Vivo Screening Imaging by Fluorescent Microscopy and Confocal Microscopy

The methods we established for in vivo time-lapse confocal microscopy were employed to monitor cellular pathologies in multiple tissues over time. Fish were returned to experimental or control conditions following imaging sessions. Subsequently, imaging frequency was adjusted to provide better temporal resolution, if deemed necessary (e.g., to resolve changes following trauma). Yellow fluorescent protein (YFP): excitation: 520 nm; emission: 546 nm. Red Fluorescent Protein (RFP): excitation: 555 nm; bandwidth: 5 nm; emission: 585 nm. Image analysis: Images were analyzed by using established ImageJ and FIJI software applications for cellular morphology studies.

### 2.6. Experimental Design: Generation of Homozygous Casper(roy^−/−^,nacre^−/−^)/myl7:RFP; Annexin-5:YFP Zebrafish (Generation F01-F06)

Each generation of the zebrafish take 3 months to achieve the adult stage [14]. Developing the new strain by cross breeding of independent strains takes 5–6 generations and the overall period is approximately 24 months.

#### 2.6.1. Screening and Sorting the Transparent Transgenic Progeny through Fluorescent Microscopy

- Breeding setup: on day 1, we selected the male and female zebrafish from a designated strain, paired them, and placed in the breeding tank supplied by Aquaneering Inc.
- On day 2, the embryos were retrieved from the bottom of the breeding tank, cleaned by removing debris, and allowed to hatch.
- On day 2, at 12 hpf stage, the embryos were cleaned by removing the dead embryos and treated with PTU media to block the pigmentation process (White et al., 2016).
- At 72 hpf stage, we carefully observed that the embryos had hatched into larvae [14]. The larvae were subjected to screening for their transgenic expression under fluorescent microscopy. The transgenic and nontransgenic larvae were separated and grouped as transgenic and nontransgenic progeny for new generation.
- The screened group of larvae were transferred into fresh fish water and allowed to hatch into further development stage.
- Larval feeding was started on 6 to 9 days post-fertilization (dpf) by maintaining daily cleaning protocol.
- At 10 dpf, the larvae were transferred into a regular fish tank and raised into the main zebrafish facility by starting hatched brine shrimp feeding from 10 to 12 dpf. After 10–12 dpf, the normal feeding was started.
- The newly generated progeny were allowed to attain the adulthood stage.
- The adult generation was subjected to breeding for next-generation F02 to F06 to achieve the larvae of homozygous strains.

#### 2.6.2. In Vivo Imaging of Homozygous Strain through Confocal Imaging

- The larvae of the newly developed homozygous strain of *Tg(/myl7:RFP;annexin-5:YFP)*; *Casper(*roy^−/−^,nacre^−/−^*)* were selected at 72 hpf stage and screened for their fluorescent expression by florescent microscopy via confocal microscopy.
- The selected larvae were mounted in 1.6% low-melt agarose, prepared by boiling and refluxing in a microwave, and then maintained at 35 °C.
- At least six larvae were anesthetized in 0.5 mL of 0.02% working media of clove oil in fish water and transferred to a 1.5 mL Eppendorf tube.
- The individual larva-contained media was added 50 ul of the 1.6% agarose solution then the larvae were gently mixed by inversion and immediately poured onto the glass-bottom microwell Petri dish (Mat Tek) (note: the larvae usually position themselves flat on their side. Thus, some larvae required gentle reorientation under the dissection scope with a pipette tip as the agarose solidified).
- As the agarose solidified, 2/3 of the petri dish was filled with fish water and the embedded larvae was ready for confocal imaging. These embedded larvae had the normal water circulation within the agarose.

## 3. Results

**Figure 2:**
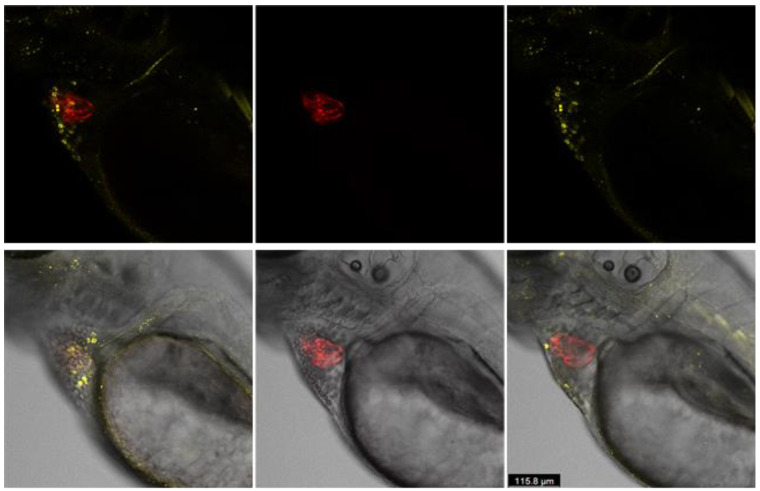
In vivo confocal imaging of the 72 hpf stage of the newly developed homozygous zebrafish larval strain of *Tg(UAS:SEC-Hsa.ANXA5-YFP,myl7:RFP)*; *Casper(*roy^−/−^,nacre^−/−^*)*. RFP (red fluorescent protein) expression shows myl7 specific to the heart, while YFP (yellow fluorescent protein) expression shows annexin-5 of the tissue and muscle area, and Casper: transparent skin.

The zebrafish provided an excellent platform to study the genetic and molecular approach of cardiac research. Zebrafish heart cells are similar to human heart cells at the molecular level, and determine gene functions that control cardiac function and dysfunction. In zebrafish heart, myl7 is a myosin light chain 7 gene, and identified as a regulatory gene ortholog to human MYL7. In the heart, annexin-5 activities contribute to cardiomyocyte dedifferentiation, proliferation, and epicardial injury responses, which leads to cardiac cell death by apoptosis and necrosis pathways. We developed annexin-5 activity in the cardiovascular function under normal and in metabolic aberration by generating homozygous Casper/myl7:RFP; annexin-5:YFP transgenic zebrafish. By developing a *Casper/myl7/Annexin-5* transparent transgenic zebrafish model, we established time-lapse in vivo confocal microscopy to study cellular phenotype/pathologies of the cardiomyocytes over time in the newly developed strain to quantify changes in cardiomyocyte morphology and function over time, comparing control and cardiac injury and cardio-oncology models. Transgenic zebrafish has normal-type skin pigmentation background. In zebrafish, tracking of transgenic reporter activity in vivo is only possible in the transparent stage. To maintain transparency throughout the life, these strains were crossbred with the skin transparent mutant *Casper(*roy^−/−^,nacre^−/−^*)*. Casper contributes to the study by integrating a transparent characteristic in adult zebrafish that allows for simpler transparent visualization and observation. We derived the stable transparent mutant *Casper(*roy^−/−^,nacre^−/−^*)*; transgenic progenies through cross breeding with the transgenic strain of *Tg(UAS:SEC-Hsa.ANXA5-YFP,myl7:RFP)*. Confocal and fluorescent microscopy were used to obtain accurate, precise imaging and to determine the fluorescent protein being activated. 1.1: Generation of homozygous *Tg(UAS:SEC-Hsa.ANXA5-YFP,myl7:RFP)*; *Casper(*roy^−/−^,nacre^−/−^*)* zebrafish (Generations F01–F06). 1.2: Screening and sorting the transgenic progeny and in vivo imaging to validate cardiac morphology through in vivo confocal imaging (Figure 2).

### Generation of Homozygous Tg(UAS:SEC-Hsa.ANXA5-YFP,myl7:RFP); Casper(roy^−/−^,nacre^−/−^) Homozygous Strain

- To develop this proposed strain, we selected six breeding pairs and crossbred Casper and myl7/annexin-5 fish strains for the progeny of F01 to F06 generation in the following order.
- F01 generation: We generated the eggs from the breeder of *Casper(*roy^−/−^,nacre^−/−^*)* mutant and *Tg(UAS:SEC-Hsa.ANXA5-YFP,myl7:RFP)* zebrafish and grew the embryo to attenuate larvae at 72 h post-fertilization (72 hpf) stage. The larvae were screened for transgenic expression. F01 generation larvae showed transgenic expression (47%), while 53% of the larval population had non transgenic expression. These larvae were allowed to grow into adult stage, and phenotypically all fish of F01 generation had normal WT skin pigmentation.
- F02 generation: The positive transgenic fishes of F01 generation were paired into male and female breeders and selected to breed for F02 generation progeny. These breeders laid the eggs, and these embryos were allowed to grow in the larvae. At the 72 hpf stage, these larvae were screened for fluorescent expression RFP (*myl7:RFP*) and YFP (*annexin-5:YFP*). A total of 39% of the F02 generation larvae showed transgenic expression. These F02 progeny were allowed to grow into adult stage, and 100% of these F02 heterozygous progeny showed normal WT skin pattern.
- F03 generation: The breeding pairs were selected from the adult breeders of the F02 generation. The F03 larval population showed transgenic expression (43%). The F03 generation progeny were allowed to grow into the adult stage, and, among the transgenic progeny, 37% of these fishes showed Casper transparent skin pattern, while 63% of the fish population expressed normal WT skin pigmentation background.
- F04 generation: To generate the F04 generation, we selected the breeders of *Casper(*roy^−/−^,nacre^−/−^*)* transgenic progeny and bred them to obtain the eggs of the F04 generation. The larvae at 72 hpf were screened for fluorescent expression to obtain transgenic strain. In F04, 90% of the larvae population showed positive fluorescent expression. In F04, 68% of fishes expressed *Casper(*roy^−/−^,nacre^−/−^*)* phenotype during growth into the adult stage.
- F05 generation: The Casper transgenic adult fishes from heterozygous transgenic progeny of F04 were paired to breed for F05 generation progeny. The larvae of the F05 generation strain were screened for transgenic expression, and 100% larvae expressed the fluorescent expression. This result confirms the homozygous progeny of the newly developed strain. These larvae of the F05 generation were allowed to grow into adults, and 100% of the F05 fish population were of Casper background, which confirms the homozygous strain genetically and phenotypically, following the Mendelian law of genetics. This strain is designated as the new line of the *Tg(UAS:SEC-Hsa.ANXA5-YFP,myl7:RFP)*; *Casper(*roy^−/−^,nacre^−/−^*)*, having the Casper transparent skin and transgenic myl7:RFP; annexin-5:YFP) expression.
- F06 generation: The F05 generation fish breeds and the new F06 progeny were homozygous by having 100% transgenic expression and 100% *Casper(*roy^−/−^,nacre^−/−^*)* transparent skin. F06 generation progeny further validated that the homozygous nature followed the Mendelian law of genetics. The newly developed strain was designated as *Tg(UAS:SEC-Hsa.ANXA5-YFP,myl7:RFP)*; *Casper(*roy^−/−^,nacre^−/−^*)^gmc1^*. The generation of this transgenic transparent homozygous strain from F01 to F06 generations is summarized in Table 1.

## 4. Discussion

The genetically engineered zebrafish model organism has been established and applied in normal basic cardiology and human congenital and acquired cardiac disease research [1]. Our main aim was to develop a transparent transgenic zebrafish model and establish time-lapse in vivo confocal microscopy to study cellular phenotype/pathologies of the cardiomyocytes over time in a newly developed zebrafish strain, to quantify changes in cardiomyocyte morphology and function over time, comparing control and cardiac injury models. We derived a stable line of zebrafish transparent mutant “*Casper(*roy^−/−^,nacre^−/−^*)* ” with the transgenic progeny of myl7/annexin-5 strain. For a visualization study of cell death process in vivo condition, the Casper transparent mutant strain was crossbred with a transgenic line expressing annexin-5-YFP fusion *Tg(UAS:SEC-Hsa.ANXA5-YFP,myl7:RFP)* [5]. In vertebrates, including murine and human systems, the in vivo spatial resolution of the adult animal is limited due to the normal opacification of skin and subdermal structures. Zebrafish falls into the same category; zebrafish embryos start to develop the skin melanophore pigmentation from 12 hpf, which continues throughout the lifespan. For in vivo imaging, the skin transparency is a primary requirement, and to maintain the transparency, blocking the pigmentation needs to be maintained. Blocking of the pigmentation can be maintained by chemical inhibition by blocking melanization. The chemical inhibition treatment is temporary and only possible until the organism is treated with the chemical inhibitor agent. The *Casper(*roy^−/−^,nacre^−/−^*)* mutant maintains transparency throughout its life and serves as the ideal combination of both sensitivity and resolution for in vivo stem cell analyses and the in vivo imaging process. To get the transparent progeny of this newly generated multiple transgenic line, different strains of zebrafish were crossbred with the *Casper(*roy^−/−^,nacre^−/−^*)* mutant [11]. Through the process of serial heterozygous and homozygous generations, we developed the multifunctional cardiomyocyte transgenic–transparent mutant line. Fluorescent microscopy was utilized to confirm the expression of fluorescent proteins in the newly generated transgenic progenies. The development of the proposed strain was designed under experimental setup in the following manner: generation of heterozygous progeny (F01, F02, F03, and F04 generations) and homozygous progeny (F05 and F06 generations); development of transparent transgenic zebrafish model and establishment of time-lapse in vivo confocal microscopy to study cellular phenotype/pathologies of the cardiomyocytes over time in a newly developed transparent transgenic zebrafish strain to quantify changes in cardiomyocyte morphology and function over time, comparing control and cardiac injury models.

## 5. Conclusions

Our main objective for this study was to develop the annexin-5 activity in the cardiovascular function under normal and in metabolic aberration, as well as during pathological circumstances by developing homozygous *Tg(UAS:SEC-Hsa.ANXA5-YFP,myl7:RFP)*; *Casper(*roy^−/−^,nacre^−/−^*)* transgenic zebrafish. This involved development of a transparent transgenic zebrafish model and establishment of time-lapse in vivo confocal microscopy to study cellular phenotype/pathologies of the cardiomyocytes over time in a newly developed transparent transgenic zebrafish strain to quantify changes in cardiomyocyte morphology and function over time, comparing control and cardiac injury models. This newly developed strain is undergoing development of the triple transgenic transparent strain by cross breeding with the NF-kB transgenic line (*Tg(6xNFκB:EGFP)nc1*) to develop a cardio-inflammatory study model. These novel results provide an in vivo whole organism-based platform to design high-throughput screening and establish new horizons for drug discovery in cardiac disease and cardio-oncology.

## Figures and Tables

**Table 1:** Summary of the newly developed strain *Tg(UAS:SEC-Hsa.ANXA5-YFP,myl7:RFP)*; *Casper(*roy^−/−^,nacre^−/−^*)^gmc1^*.

Progeny	Strain	Transgenic Expression		Skin Pigmentation Background Pattern	
		Transgenic Expression *myl7:RFP/ANXA5-5:YFP*	Nontransgenic Expression	*Casper(*roy^−/−^,nacre^−/−^*)* Transparent Skin Pattern	Normal WT Skin Pigmentation
F01 Generation	Heterozygous	47%	53%	0.0%	100%
F02 Generation	Heterozygous	39%	61%	0.0%	100%
F03 Generation	Heterozygous	43%	57%	37%	63%
F04 Generation	Heterozygous	90%	10%	68%	32%
F05 Generation	Homozygous	100%	0.0%	100%	0.0%
F06 Generation	Homozygous	100%	0.0%	100%	0.0%

## Data Availability

The original data presented in this study can be obtained from authors upon request.
